# Animal health syndromic surveillance system in Jordan, a road map for a pilot model

**DOI:** 10.3389/fvets.2025.1538347

**Published:** 2025-05-30

**Authors:** M. De Nardi, S. Küker, S. Salah, I. Qtananni, F. Rosso, S. Baiomy

**Affiliations:** ^1^SAFOSO AG, Liebefeld, Switzerland; ^2^Department of Veterinary Medical Sciences, University of Bologna, Bologna, Italy; ^3^Early Warning & Risk Assessment Department, Veterinary & Animal Health Directorate, Ministry of Agriculture, Hamman, Jordan; ^4^Veterinary & Animal Health Directorate, Ministry of Agriculture, Hamman, Jordan; ^5^European Commission for the Control of Foot-and-Mouth Disease, Food and Agriculture Organization of the United Nations, Rome, Italy

**Keywords:** surveillance, syndromes, syndromic surveillance system, EuFMD, early warning system, Jordan

## Abstract

**Introduction and objectives:**

Early detection of transboundary animal diseases (TADs) is critical to mitigating economic losses and safeguarding food security. These diseases pose significant threats to regions including North Africa, the Near East, and Southeast Europe. Recognizing the importance of early detection, the European Commission for the Control of Foot-and-Mouth Disease, in collaboration with SAFOSO AG, launched a multiphases project to introduce syndromic surveillance system (SyS) in the Near East, with Jordan eventually selected as the pilot country. In Jordan, therefore, the project aimed to enhance disease surveillance capabilities through the design and implementation of a pilot SyS.

**Project’s elements:**

Initially, a scoping review of global SyS initiatives was conducted, with a specific focus on North Africa, Southeast Europe and the Near East. This was followed by a regional workshop in Beirut, Lebanon, where SyS concepts were introduced to regional stakeholders. During this workshop Jordan was selected for the SyS pilot study. The final phase involved the development of a tailored pilot SyS in Jordan which included extensive stakeholder engagement through workshops and virtual follow-ups. Key technical activities encompassed syndrome definition, evaluation of data availability and data access, selection of appropriate statistical methodology. Governance was established through the formation of steering and technical committees, and supported by a communication strategy for effective dissemination of findings. A road map was developed to monitor the pilot syndromic surveillance initial implementation and long-term progresses.

**Results:**

The pilot SyS in Jordan was designed to integrate diverse data sources, including clinical and laboratory reports, slaughter statistics, livestock production data, public health information and animal movement records. Designed to prioritise early detection, it included comprehensive data collection, rigorous analysis, and systematic dissemination. The design of this pilot SyS in Jordan highlights its feasibility and benefits for early disease detection, driven by strong stakeholder engagement, legal framework alignment, and robust information technology infrastructure.

**Conclusion:**

The successful implementation of the pilot SyS in Jordan underscores its potential as a model for other countries in the region. The project has enhanced capacity for disease surveillance, supporting TADs control and prevention. Sustained collaboration, capacity development, and monitoring will be essential for scaling the SyS at national level and ensuring long-term success in combating transboundary animal diseases.

## 1 Introduction

In the current Anthropocene epoch, global warming, livestock intensification, biodiversity loss, human population growth and increased animal-human interaction escalate the risk of (re)emerging diseases. Early detection of infectious diseases is vital for safeguarding animal and public health (1–5). Transboundary diseases like foot-and-mouth disease (FMD) carry major economic impacts due to globalization and intensive farming. FMD outbreaks cause trade restrictions, production losses, and market disruptions, emphasizing the need for robust detection and control measures.

In recent years, the mandate of the European Commission for the Control of Foot-and-Mouth Disease (EuFMD) has been expanded beyond risk reduction of only FMD to encompass similar transboundary animal diseases (5) (summarized under the term “FAST” diseases), including Rift Valley fever (RVF), peste des petits ruminants (PPR), capripoxviruses [sheep and goat pox (SPGP) and lumpy skin disease (LSD)] in order to address emerging disease threats in North Africa, the Near East and Southeast Europe (6).

FAST diseases result in significant animal and public health impacts, with profound social and economic implications (7–9). An efficient, cost-effective early warning system is crucial to enable a country to respond swiftly to disease introductions, and mitigate long-term consequences (10). Such systems rely on high quality data generated through timely and highly sensitive surveillance systems. Traditionally, veterinary surveillance targets single diseases. However, the changing landscape of possible health threats, and limited resources, make this approach unsustainable. The integration of information from multiple data sources enhances early warning systems, improving their capacity to detect health events promptly (11).

Syndromic surveillance (SyS) systems utilize multiple sources of data to detect potential human and/or animal health threats, enhancing early detection and early warning for FAST diseases. Syndromic surveillance is defined as a real-time (or near real-time) collection, analysis, interpretation and dissemination of health-related data that enables rapid identification of public or veterinary health risks requiring action (12). Over the past two decades, SyS methodologies have advanced significantly, driven by innovations in information technology and disease informatics, including data mining, automated analysis systems and artificial intelligence algorithms (11, 13).

Syndromic surveillance aims to detect outbreaks earlier than would otherwise be possible with laboratory-based methods by utilizing timelier, though less specific, data sources (13). It complements, but does not replace, laboratory-confirmed surveillance, which remains essential for confirming suspected outbreaks signaled by SyS. Case definitions used for SyS are data source-dependent categories, focusing on clinical signs rather than on specific laboratory-confirmed causative agents. This lack of specificity allows the system to target general disease groups, or syndromes, hence the term “syndromic surveillance.” These systems were developed to make use of pre-diagnosis data already available and automatically collected, such as chief complaints upon emergency visit, necropsy data, livestock performance data or laboratory test orders (13–16).

Recent inventories of SyS initiatives in public health have been conducted (17–21). However, reviews of SyS in animal health remain limited and mostly focused on the methods applied presenting therefore only a partial view (11, 13, 22). To address this gap, the authors conducted a scoping review (23) on global veterinary SyS initiatives for livestock, with emphasis on North Africa, Southeast Europe and the Middle East, between 2016 and 2023. Most references reviewed focused on potential data sources for SyS, rather than SyS initiatives implemented in specific countries. Examples of multivariate data source syndromic surveillance systems include the National Cattle Health Surveillance System in the Netherlands (24), the Canada West Swine Health Intelligence Network (25), the Ontario Animal Health System (26) and the Veterinary Investigation Diagnosis Analysis database in Great Britain (27). Additionally, a SyS system focusing on bovine mortality data (Observatoire de la Mortalité des Animaux de Rente OMAR), is active in France (28). No scientific literature was identified that described active SyS systems or initiatives in Southeast Europe, North Africa or the Middle East.

## 2 Overall objectives and geographical context of the project

Under its Move-FAST strategy (29), the EuFMD aims to promote the use of SyS in countries within the European neighborhood to improve the early detection of, and response to, FAST diseases. In collaboration with SAFOSO AG, EuFMD initiated a pilot project to introduce SyS in the Near East, with Jordan selected for a case study, considering lessons learnt from SyS initiatives in other countries.

This manuscript describes the project’s main programmatic elements and key outputs, focusing on the design of the pilot SyS system in Jordan. We then discussed the practical implications of the SyS system and lessons learned for future applications.

## 3 Programmatic elements

The project was executed in three phases. Initially, a scoping review of global SyS initiatives was conducted, with a specific focus on North Africa, Southeast Europe and the Near East. The results of the scoping review are described in Küker and De Nardi (23), under publication. The review was followed by a regional workshop in Beirut, Lebanon, where SyS concepts were introduced to regional stakeholders, ultimately leading to the selection of Jordan for the pilot study. The final phase involved the development of a tailored pilot SyS in Jordan, which included extensive stakeholder engagement through workshops and virtual follow-ups.

### 3.1 Regional workshop in Beirut

A regional two-day workshop was held in Lebanon in May and June 2023. The workshop gathered professionals working in competent authorities from Jordan, Palestine, Libya, Lebanon and the Syrian Arabic Republic. Egypt and Sudan were invited but unable to attend.

The main objectives of the workshop were (i) to introduce the concept and methods for syndromic surveillance, (ii) to discuss the feasibility, strengths, limitations and benefits of adopting and integrating a syndromic surveillance system into the current national surveillance efforts and (iii) to identify a country in which a pilot SyS for FAST diseases could be designed.

Participating countries were asked to provide specific information through a questionnaire, and additional information was discussed during the workshop. The questionnaire gathered the following information:

- The country’s current legal framework for surveillance programs, including SyS;
- A description of existing surveillance systems, including stakeholders, data sources, data transmission mechanisms, data storage and databases, data analysis approaches, and reporting mechanisms;
- A description of existing animal and public health healthcare infrastructures;
- A description of potential partners from both the public and private sectors who could support a SyS.

The discussion focused also on possible data sources for a SyS targeting FAST diseases in interested countries, as well as the data management and analysis capabilities required. Jordan was selected to design and implement a pilot SyS system in the region (23). The presence of a conducive legal framework, data accessibility and quality, presence of adequate information technology infrastructure, institutions and stakeholder presence and support were relevant criteria for the selection. Information gathered through the questionnaires, the discussions during and after the workshop in Beirut, further highlighted Jordan’s suitability for the pilot SyS system. Additional elements such as the ongoing collaboration between public and veterinary health sectors, and their strong motivation to contribute to the SyS system development collectively made Jordan the ideal choice for the pilot project.

### 3.2 Pilot project in Jordan

Through two workshops and virtual follow-up meetings, Jordanian stakeholders were guided through the necessary steps to design a SyS tailored to the conditions in the country. The motto from the Triple-S guideline “Think big, start small, and get the key people on board” inspired the activities in Jordan (30). Considering the task’s complexity, the team set realistic expectations of what the system could and could not deliver. Consequently, the stakeholders agreed that the prototype SyS should consider a single production sector (i.e., the dairy sector) in specific regions (i.e., the two regions in Jordan where this sector is most relevant). The upscale of the pilot SyS at national level and its expansion to different production sectors is one of the key actions, in the long term, in the roadmap presented in the chapter 3.2.5.

The workshops were delivered in Jordan in July and September 2023, and gathered professionals working with the veterinary competent authorities alongside potential stakeholders. The recent (January 2023) FMD epidemics in Jordan have had a significant impact on the dairy sector. As a consequence, due to the epidemics, there were already meetings and collaborations between the public veterinary competent authorities and other private and public stakeholders in place. This initial coordination and communication among key stakeholders were considered a promising foundation for the SyS.

Table 1 shows the programmatic steps deployed in Jordan to develop the pilot SyS based on the Triple S guidelines (30), and the rationale for each step. The table include also few programmatic elements not implemented during the project, due to time constraints, but that are necessary to fully establish a SyS.

**Table 1:** Programmatic steps for the development of the SyS in Jordan.

Key steps implemented in Jordan	Rationale (why each step is important)
Evaluation of the epidemiological situation in the country with regards to hazards of concern, surveillance mechanisms and livestock production sector.	Crucial for understanding the current risks and vulnerabilities within the country’s livestock sector, which informs the development of targeted SyS and intervention strategies.
Review of specific functions of the country’s competent veterinary authority, in collaboration with those competent authorities.	Important for determining gaps in the current functioning of surveillance programs. This will help to define the overall objectives and scale of the SyS.
Evaluation of the legal framework under which a SyS will be implemented.	Understanding the legal framework is essential to ensure that the SyS operates within existing laws and regulations, and to identify any necessary changes or enhancements required for effective implementation.
Evaluation of the suitability of the national information technology environment and existing data management systems.	Important for assessing whether the current IT infrastructure can support the SyS requirements, including data collection, storage, access and analysis. This will help identify any upgrades or adjustments needed for optimal system performance.
Definition of the SyS objectives in relation to the animal production and species targeted.	Clear objectives are necessary to focus the SyS on relevant animal production sectors and species, ensuring that the system effectively addresses the specific needs and risks associated with these areas
Identification of main stakeholders and partners of SyS.	Crucial for fostering collaboration, ensuring that all relevant parties are involved in the development and implementation of the SyS, and facilitating effective communication and resource sharing.
Identification of data sources and data providers.	Crucial for ensuring that the SyS has reliable and comprehensive data inputs. This step helps in establishing a network of information flow, ensuring that all relevant data needed for surveillance is accessible and integrated into the system for accurate analysis and timely response.
Definition of “syndrome”: Specifying a set of clinical signs or patterns of illness that can indicate particular disease(s) or condition(s).	This step is important for standardizing the criteria used to detect and report potential outbreaks, ensuring consistency in surveillance and enabling effective monitoring and response across different regions and stakeholders.
*Development of a statistical algorithm to validate data and generate an alert from the SyS.	Essential for processing, analyzing and validating data, and generating timely alerts for disease outbreaks.
*Definition of a communication strategy.	Important for ensuring that information about the SyS, including alerts and updates, is effectively disseminated to stakeholders, partners and the public, facilitating coordinated response.
Create a management structure for the SyS and define governance issues.	Critical for ensuring that the SyS operates efficiently, with clear roles and responsibilities, and that it adheres to established protocols and standards.
Development of a roadmap to launch the pilot SyS and scale it to a national level.	A roadmap provides a strategic plan for implementing the pilot SyS and scaling it nationally, ensuring that the system is introduced systematically and expanded effectively to cover all necessary regions.
*Define criteria and plan to regularly evaluate the performance of the system.	Regular evaluation helps monitor the effectiveness and efficiency of the SyS, allowing for continuous improvement and adaptation based on performance outcomes and emerging needs.

*Steps not implemented in the project, but necessary to fully establish a SyS.

#### 3.2.1 The institutional and epidemiological context in Jordan

In Jordan, the Ministry of Agriculture (MoA) oversees the veterinary sector through the Animal Health Department, which manages veterinary surveillance strategies and the Electronic Integrated Disease Surveillance System (EIDSS). The EIDSS integrates various data sources in a standardized format, enabling animal health monitoring. Therefore, the country has a foundational informational technology infrastructure in veterinary medicine, which supports electronic data storage and access that might be functional to a SyS.

Jordan’s veterinary competent authority had prioritized several hazards, including FMD, PPR, SPGP, camel pox, anthrax, LSD, bovine tuberculosis, rabies, avian influenza and brucellosis. A severe FMD outbreak in Zarqa and Mafraq regions in January 2023, with thousands of cases, led to a nationwide market closure affecting trade with neighboring countries and caused significant economic damage. Outbreaks of PPR and LSD have been reported in 2006 and 2013, respectively. Authorities described delays in reporting and investigating these outbreaks due to specific challenges related to surveillance design, financial resources and partnership between the public and private sectors.

#### 3.2.2 Objectives of pilot SyS, target production type/location, key stakeholders

The pilot SyS aimed to contribute to early detection of FAST diseases and other relevant animal infectious diseases in Jordan, including some public health concerns. Syndromes were identified that would raise alerts for the following hazards, in the targeted livestock production sector (commercial dairy farms): FMD, LSD, RVF, brucellosis, anthrax, rabies, infectious bovine rhinotracheitis, ephemeral fever and bluetongue. However, due to the non-specific nature of SySs, it is acknowledged that other diseases with similar clinical patterns would also be detected (and alerts generated).

The pilot SyS targeted commercial dairy farms in the Zarqa and Mafraq regions. These regions are currently holding approximately 65% of the total number of commercial dairy cattle farms in Jordan (40% in Zarqa, 25% in Mafraq). Jordan’s Veterinary and Animal Health Directorate contains an Early Warning and Risk Assessment department, which was designated to oversee the implementation and future development of the pilot SyS. Key stakeholders, including the National Livestock Association, Private Veterinary Association, Greater Amman Municipality Slaughterhouse, and the Jordan Center for Disease Control (JCDC), confirmed their support for future development and implementation.

A detailed list of potential stakeholders identified, their responsibilities, and the engagement plan is provided in Supplementary Table 1.

#### 3.2.3 Potential data sources, data providers and data management system

Specific data sources for the pilot SyS were selected by the stakeholders and are shown in Supplementary Table 2. These included slaughter statistics, clinical information, livestock production data, treatment information and sales data. Additional data (livestock movement, trade data) that were not accessible could represent a future source of relevant information. Each data source was assessed for accessibility, data quality, representativeness, validity and timeliness. These aspects are critical, as the underlying statistical algorithm of the SyS that will generate alerts related to potential outbreaks is highly dependent on data quality.

The Electronic Integrated Disease Surveillance System, developed under the Biological Threat Reduction program (BTRP), launched in Jordan in 2021 and funded by the US Defense Threat Reduction Agency (DTRA), integrates veterinary and human disease surveillance data. This system was selected by the stakeholders as the data management systems for the pilot SyS. Currently, over 160 official veterinarians provide data to EIDSS. The Central Animal Health Laboratory provides laboratory data to EIDSS, including species, clinical signs and diagnostic test results. EIDSS is currently implemented in 13 slaughterhouses under the Ministry of Local Administration and Great Amman Municipality, whose veterinarians report syndromes on EIDSS on a potentially daily basis.

In this way, EIDSS offered valuable, standardized electronic data, and could be adapted to gather additional data streams (including the ones selected for the pilot SyS). However, staffing shortages and connectivity issues can impact the timeliness of reporting.

#### 3.2.4 Syndrome definition and data quality considerations

A syndrome is a group of clinical signs that suggest the presence of a disease or condition before it is confirmed. Syndromes can either be specific to a particular condition or consist of non-specific clinical signs. Given that single clinical signs are often non-specific, it is helpful to define a combination of them to improve the specificity of a syndromic surveillance system. A “sensitive” definition effectively identifies the presence of a syndrome, reducing false negatives, while a “specific” definition identifies its absence, reducing false positives. The choice between sensitivity and specificity depends on the severity of the syndrome and the importance of minimizing false outcomes.

In Jordan, specific criteria were developed to select relevant data streams for SyS. These criteria included ensuring each disease was represented by at least two clinical signs and that each data type was relevant to at least two hazards (Supplementary Table 3). There were some exceptions identified, for example, “lesions on the hooves and in the mouth,” critical clinical signs for FMD, were included in the selection despite that these only refer to FMD.

Data source quality and reliability are essential, and gaps should be addressed by integrating additional data sources, such as pharmacy sales and animal movement data. Pharmacy sales data can provide insights into trends in medication purchases, potentially signaling outbreaks associated with specific clinical signs, while animal movement data is vital for understanding spatial pathways of disease transmission. Integrating these data sources enhances the system’s ability to detect and respond to health events effectively.

The final inclusion of data streams should consider accessibility, quality, and the potential contribution to the SyS. Supplementary Table 3 shows the list of suitable data types (referring to both direct and indirect clinical signs) in Jordan, and the relevance of each data source/type for each prioritized hazard (the syndrome) in cattle.

Data analysis in the SyS, a task requiring statistical or epidemiological expertise, aims to produce credible outputs that align with the system’s objectives. Consistent data quality is crucial because fluctuations will impact the system’s accuracy. Factors such as inconsistent diagnostic coding, lack of data from certain geographic areas, or personnel turnover can cause these fluctuations and should be addressed.

To ensure SyS’s credibility, it is vital to control data quality. This can be achieved by maintaining a data quality log to track problems in the dataset. Examples of data quality checks include monitoring the number of records received, the structure and completion rate of data fields, and redundancy checks through overlaps in data uploads. These measures help ensure that the syndromic surveillance system remains reliable and effective in monitoring and identifying health trends.

#### 3.2.5 A roadmap for SyS development

The development of a detailed roadmap was crucial in guiding the initial implementation of the SyS in Jordan. The importance of structured planning in the successful deployment of surveillance systems is consistently underscored in the literature (30).

The roadmap was structured into main components (legal, operational, governance, capacity building, evaluation, upscaling at the national level). For each component, the following aspects were agreed: main action, level of priority of the action, timeframe for the action, responsible stakeholder for the action, possible stakeholder partner for the action, steps required to fulfil the action. A total of 19 actions were agreed. Table 2 shows, as an example, the two main actions, with regard to data streams and syndrome definition, considered necessary under the operational component of the roadmap. The complete Roadmap is shown in Supplementary Table 4.

**Table 2:** Section of the roadmap to implement the SyS in Jordan.

Main action	Level of priority	Timeframe	Responsible stakeholder	Partner stakeholder	Steps to fulfil the action
1. Confirm data streams to detect syndromes	Urgent	Medium	Veterinary and Animal Health Directorate	JCDC/University (JUST), external consultants	Perform first statistical evaluation of identified data streams Prioritize data streams/syndromes
2. Development of analytical algorithm (SyS statistical model)	Urgent	Medium	Veterinary and Animal Health Directorate	JCDC/University (JUST), external consultants	Define terms of reference of SyS Statistical model Develop and test the SyS stat model

## 4 Discussion

This study documents the development and initial implementation of a SyS in Jordan. This approach can also serve as a model for other countries aiming to strengthen their animal disease surveillance capabilities. The experiences gained from this pilot project provide valuable insights into key aspects of SyS implementation.

### 4.1 Key aspects for SyS implementation

Implementing the SyS in Jordan revealed several critical factors that align with findings in the existing literature. One of the primary challenges was ensuring the validation and integration of diverse data streams. Previous studies have highlighted that data quality and consistency are fundamental to the success of any surveillance system (13, 14). In Jordan, incomplete data entries and inconsistent reporting formats were identified, which are common challenges in similar surveillance initiatives globally (26, 27). Addressing these gaps required careful attention and a coordinated approach among stakeholders, reflecting the requirement for robust data management frameworks (11).

Strong stakeholder engagement was a key enabler, consistent with descriptions that emphasize the importance of involving a wide range of participants in surveillance systems (22). The success of the SyS in Jordan hinged on the active participation of government authorities, private sector representatives and local farmers. Building trust and ensuring clear communication among these stakeholders is vital for achieving a cohesive and functional surveillance system (21). This aligns with the participatory approach taken in Jordan, where key stakeholders identified were involved at every stage, from system design to implementation (Supplementary Table 1).

The availability of a detailed roadmap was an important tool to drive the initial implementation of the SyS in Jordan. The roadmap provided a phased approach that allowed for systematic progress while accommodating Jordan’s unique context and needs. Starting with a focus on specific regions and production sectors, such as the dairy sector in Zarqa and Mafraq, allowed the project to build momentum gradually. This type of phased implementation has been successful for other surveillance systems (24).

Flexibility, enabling changes based on ongoing feedback and emerging challenges, is also reflected in the literature, emphasizing the need for adaptability in surveillance systems (15). A flexible approach not only ensured that the SyS in Jordan was well-tailored to local conditions but also facilitated the necessary training and capacity development efforts that are required for long-term sustainability.

### 4.2 Potential for replication in other countries

The approach taken in Jordan offers a valuable template for other countries, particularly those with similar socioeconomic and technical contexts. The structured roadmap, with its emphasis on phased implementation and stakeholder engagement, aligns with best practices. These include leveraging existing data sources, automating processes for data extraction and analysis, ensuring data quality, securing data handling, fostering stakeholder collaboration, defining clear objectives, and planning for long-term sustainability (30). It is possible to overcome common challenges in SyS implementation, such as data fragmentation, balancing timeliness and accuracy, resource limitations, standardization of definitions, validation and interpretation of data, building trust, and ensuring sustainability and scalability (20).

The validation and final selection of the data streams identified as potential data sources will represent a significant challenge; some already present limitations (Supplementary Table 2). There are opportunities to improve the existing surveillance systems in Jordan, and this would facilitate data entry, consistent reporting and proper data integration. The variation in technical infrastructure across regions further complicated the validation process, necessitating extensive collaboration and cross-checking with local stakeholders.

The development of a reliable analytical algorithm is another critical component that requires specific technical expertise. The complexity of creating an algorithm capable of accurately detecting syndromes from noisy data, compounded by the lack of historical data for training, is a significant challenge. The algorithm has to be flexible, adaptable to local disease contexts, and continuously refined as the system expands. The need for advanced computational expertise at the national level is key and should be addressed with ongoing investment in both technical infrastructure and expertise.

For these reasons, the roadmap has identified clear actions (Table 1).

By sharing the methodologies and lessons learned from the Jordan pilot, this project contributes to broader regional and global efforts to enhance animal health surveillance. The roadmap can be adapted and allow other countries to tailor the approach to their specific needs, ultimately strengthening global health security. As the literature suggests, such replication of successful models can lead to significant improvements in early detection and response to transboundary animal diseases (19).

## Data Availability

The original contributions presented in the study are included in the article/Supplementary material, further inquiries can be directed to the corresponding author.
