# Supplementary material for: Animal health syndromic surveillance system in Jordan, a road map for a pilot model
Source: Front Vet Sci. 2025 May 30;12:1538347. doi: 10.3389/fvets.2025.1538347 (PMC12162469; doi:10.3389/fvets.2025.1538347)
Supplement: Supplementary file 1 [file Table_1.docx]

Supplementary Material

# Supplementary Tables

**Supplementary Table 1: Stakeholders and responsibilities**

Table 1 shows the potential stakeholders of the SyS in Jordan with a description of their responsibilities and a plan to engage them. The table represents the outputs of specific working group session implemented in Jordan during two workshops. The project team has not yet met all potential stakeholders listed so not all the stakeholders in Table 1 have confirmed their interest (e.g., Universities and academia). However, they are listed in the table because they are considered potential relevant partners in the future SyS.

| **Key stakeholder** | **Role and responsibility** | **Ideas for engagement** | **Department or institution responsible** | **Timeframe for engagement** |
| --- | --- | --- | --- | --- |
| Animal Health Department | Responsible for SyS implementation and further development | - develop notifiable syndromes list - implementation of veterinary syndromic surveillance module on the Electronic Integrated Disease Surveillance System (EIDSS) | VAHD | 1 year |
|  | Definition of syndromes | - establishment of regulations committee - EIDSS regulations - priority disease regulations |  | 2-5 years |
|  | Review and enforcement of regulations | - legal empowerment of the CVO role - activation of EIDSS follow up committee to attain sustainability |  | 2-5years |
|  | SOPs for disease notification and intervention | - development of reporting SOPs - fix gaps on EIDSS alert system - development of communication strategy between stakeholders - training of field vets on the developed SOPs - develop animal disease surveillance plan |  | 1 year |
|  | Issuing reports (communication) and data analysis | - advance training on Epidemiological analysis, interpretation and reporting of data - expertise is necessary to define detection threshold based on local epidemiological contest |  | 1 year |
|  | Training and capacity building | - Continuous training - Advocate and improve awareness on importance of animal disease surveillance - EIDSS sustainability |  | 1-3 years |
| Private veterinarians | Immediate notification | regular meetings | VAHD | 2-3 years |
|  | Weekly report | training programs concerning surveillance and disease control purposes |  | 2-3 years |
|  | Immediate intervention | development of an agreement on data sharing |  | 2-3 years |
| Private Veterinary Association | - Support in law development related to good veterinary practice - Interface for communication between the private and public sector - Join and share data related to ruminant health | - develop and implement new regulation in accordance with Agri law and international standards | Private Veterinary Association | 1-5 years |
| National Livestock Association | Communication to farmers and regular reporting | - Support the system among their members - Enforce electronical data collection and sharing | NLA | 1-5 years |
| Slaughterhouse veterinarians (Municipality) | Report of notifiable diseases | - regular meetings - joint trainings - collaboration agreement - EIDSS | - slaughterhouses - department municipalities | 1-2 years |
|  | Report of condemnation rates and reasons | - awareness of existence of Syndromic system - spontaneous reporting - EIDSS |  | 1-2 years |
| Drug stores | Sharing data of condemnation rates, slaughter statistics and meat inspection | - meetings - vet association cooperation - conduct syndromic surveillance concept workshops - development of regulations | Pharmaceutical and Drug Control Department | 1-5 years |
|  | Report sales of antibiotic and antipyretic medications |  |  |  |
| Farmers | Reporting notifiable diseases and agreed data | - increase awareness about the importance of reporting syndromes - awareness trainings, Brochures, media etc - mobile reporting applications | - VAHD - veterinarians in agricultural directorates | 1-3 years |
| International organizations | Financial support | - financial support for implementing different activities | donors | 1-2 years |
| IT experts | Programmers | - mobile reporting applications | - Private sectors - Digital Transformation Directorate (MOA) | 1-2 years |
| Feed and Identification Directorate | Data provision of animal identification and traceability | - Electronic Identification System /EIDSS | - VAHD - Feed &identification Directorate | 2-5 years |
| Animal Wealth Laboratories Directorate (AWDL):   - pathology unit of the Central Veterinary Laboratory - peripheral laboratory units in the governorates | Reporting of laboratory requests | - EIDSS (lab module) - activation of the role of the lab on the EIDSS - activation of the role of labs on EIDSS follow up committee - trainings on the concept of Syndromic surveillance | - VAHD - AWLD | 1-2 years |
| - Public field veterinarians - Veterinarians in Agriculture Directorates - veterinarians in veterinary quarantine and border agriculture centres | Reporting of investigations, follow ups and interventions | - data sharing on EIDSS / continuous trainings | - Agriculture Directorates - VAHD | 1 years |
| Universities and academia expertise | Epidemiological data analysis | - regular meetings - consultancy - workshops | - VAHD - Universities | 2-3 years |
| Animal Production Directorate | Reporting on production data and farm distributions | - workshops - regular meetings - data sharing | - VAHD - Animal production directorates | 1-2 years |
| Jordan CDC | Report of zoonotic diseases in humans  Supervision over the animal health situation and data analysis expertise | - EIDSS view - Workshops - regular meetings | - VAHD - Animal wealth laboratories - JCDC | 1-3 years |
| RSCN (wildlife) | Wildlife reports (e.g., mortality rate) and notification to the veterinary directorate | - joint meetings - workshops | - RSCN - VAHD - Agricultural Directorates | 1-3 years |
| MOH | Notification about zoonotic diseases (human cases) caused by suspected animal source | - Joint meetings - workshops - one health committee | - VAHD - Zoonotic department - MOH | 1-2 years |

**Supplementary Table 2: Data Sources**

The table shows the type of information, the data, its provider and estimated accessibility. Furthermore, the table indicated the necessary steps to access the data, the department or institution responsible and an estimated timeframe for the steps. The selection is preliminary and the final selection can only be made after the different data are checked for its quality and accountability.

| **Type of information** | **Data** | **Data provider** | **Accessibility** | **Necessary steps to access data** | **Responsibility** | **Timeframe for steps** |
| --- | --- | --- | --- | --- | --- | --- |
| Clinical information | Diarrhea | Veterinarian, farmer, RSCN | Easy | - reporting - passive surveillance - Electronic Integrated Disease Surveillance System (EIDSS) | Veterinarians | 1-2y |
|  | Fever |  |  |  |  |  |
|  | Lameness | Veterinarian, farmer |  |  | Veterinarians, drugstores |  |
|  | Lesions on foot and mouth | Slaughterhouse, veterinarians, farmers |  |  | Veterinarians |  |
|  | Mastitis | Veterinarians, farmer |  |  | Veterinarians, farmer |  |
|  | Neurologic signs |  |  |  |  |  |
|  | Skin lesions |  |  |  |  |  |
|  | Hemorragies |  |  |  |  |  |
|  | Orchitis |  |  |  |  |  |
|  | Fertility |  |  |  |  |  |
|  | Respiratory signs |  |  |  |  |  |
|  | Retended placenta |  |  |  |  |  |
| Pharmacy sales | Increase of vaccination | Drug store, veterinarian, farmer | Difficult | obligatory official monthly report | Veterinarians |  |
|  | Increase of AB-sales |  |  |  |  |  |
|  | Increase of antipyretic drug consumption |  |  |  |  |  |
| Production information | Increase of abortions | Farmers, veterinarians | Easy | - Passive surveillance - EIDSS | Veterinarians |  |
|  | Increase of mortality rate |  |  |  |  |  |
|  | Decrease of milk yields |  | Difficult | Implementation of privacy to enhance collaboration | Farmer |  |
|  | Disposal rate |  |  |  |  |  |
|  | Decrease of feed consumption |  |  |  |  |  |
| Slaughter statistics | Increase of condemnation rates | Slaughterhouses under the authority of municipalities |  | - enforcement of Agri law - collaboration agreement to ensure monthly reporting of condemnation rates and related health issue - accelerate implementation of EIDSS in the slaughterhouses - establishment of follow up committee for reporting on EIDSS that include members from VAHD and slaughterhouses - joint regular meeting with VAHD and slaughterhouse in municipalities | Slaughterhouse department, CVO |  |
| Public Health information | Human cases | JCDC, MOH |  | Establishment of general data sharing agreements on zoonotic diseases cases in humans | AHD, JCDC, MOH |  |
|  | Bite history |  |  |  |  |  |
| Animal movement | Animal trade (inter and external) | MoA | Easy | Full and functional implementation of the animal identification program | MoA, AHD |  |
| Laboratory information | Laboratory requests | Animal Wealth Laboratory |  | EIDSS notification | AHD |  |
| Export of goods | Decrease of hides export | MoA | Easy | Monthly report | MoA, AHD |  |

AHD: Animal Health Department; EIDSS: Electronic Integrated Disease Surveillance System; JCDC: Jordan Center of Disease Control; MoA: Ministry of Agriculture; MOH: Ministry of Health;

**Supplementary Table 3: Data sources and relevance for FAST diseases**

Supplementary Table 3 shows the list of suitable data source and types (referring to both direct and indirect signs) in Jordan, and the relevance of each data sources/type for each prioritized hazards in cattle. For instance, respiratory signs are relevant signs for both IBR and TB (identified as ✓ in the table). As indirect sign, e.g., animal movement could be relevant as diseases outbreaks could lead to increased trade of asymptomatic animals before disease control measures are potentially leading to financial losses to the animal owner after case notification.

| **Information** | | **Disease** | | | | | | | | | |
| --- | --- | --- | --- | --- | --- | --- | --- | --- | --- | --- | --- |
| **Type of information** | **Detailed information** | **Rift valley fever** | **FMD** | **Lumpy skin** | **Anthrax** | **Brucellosis** | **TB** | **IBR** | **Ephemeral fever** | **Rabies** | **Total overlap** |
| **Direct signs** | | | | | | | | | | | |
| Clinical information | Lesions on foot and mouth | - | ✓ | - | - | - | - | - | - | - | 1 |
|  | Respiratory signs | - | - | - | - | - | ✓ | ✓ | ✓ | - | 3 |
|  | Diarrhea | ✓ | - | - | - | - | ✓ | - | - | - | 2 |
|  | Fever | ✓ | ✓ | ✓ | - | - | ✓ | ✓ | ✓ | - | 6 |
|  | Lameness | - | ✓ | - | - | - | - | - | ✓ | - | 2 |
|  | Mastitis | - | ✓ | - | - | ✓ | - | - | - | - | 2 |
|  | Neurologic signs | ✓ | - | - | - | - | - | - | - | ✓ | 2 |
|  | Skin lesions | - | - | ✓ | - | - | - | - | - | - | 1 |
|  | Hemorragies | ✓ | - | - | ✓ | - | - | - | - | - | 2 |
|  | Orchitis | - | - | - | - | ✓ | - | - | - | - | 1 |
|  | Fertility | - | - | - | - | ✓ | - | - | - | - | 1 |
|  | Retended placenta | - | - | - | - | ✓ | - | - | - | - | 1 |
| **Indirect signs** | | | | | | | | | | | |
| Pharmacy sales | Increase of vaccination | - | ✓ | ✓ | ✓ | ✓ | - | ✓ | - | - | 5 |
|  | Increase of AB consumption | ✓ | ✓ | ✓ | ✓ | ✓ | - | ✓ | ✓ | - | 7 |
|  | Increase of antipyretic drug consumption | ✓ | ✓ | ✓ | - | - | - | ✓ | ✓ | - | 5 |
| Production information | Decrease of feed consumption | ✓ | ✓ | ✓ | - | - | ✓ | ✓ | ✓ | - | 6 |
|  | Increase of Abortions | ✓ | - | ✓ | - | ✓ | - | ✓ | - | - | 4 |
|  | Increasing Mortality rate | ✓ | ✓ | ✓ | ✓ | - | - | - | - | - | 4 |
|  | Decreasing Milk yields | ✓ | ✓ | ✓ | - | - | - | ✓ | ✓ | - | 5 |
| Slaughter statistics | Increase of condemnation rates | - | - | - | - | - | ✓ | - | - | - | 1 |
| Public Health information | Human cases | ✓ | - | - | ✓ | ✓ | ✓ | - | - | ✓ | 5 |
|  | Bite history | - | - | - | - | - | - | - | - | ✓ | 1 |
| Production information | Disposal rate | ✓ | ✓ | - | **✓** | - | - | - | - | - | 3 |
| Animal movement | Animal trade (inter and external) | ✓ | ✓ | ✓ | ✓ | ✓ | - | - | - | - | 5 |
| Laboratory information | Laboratory requests | ✓ | ✓ | ✓ | ✓ | ✓ | ✓ | ✓ | ✓ | ✓ | 9 |
| Export of goods | Decrease of hides export | - | - | ✓ | ✓ | - | - | - | - | - | 2 |
| Sum of selected signs for each disease | | 7 | 7 | 6 | 5 | 5 | 6 | 6 | 5 | 2 |  |

**Supplementary Table 4: Roadmap**

| **Code** | **Main action** | **Level of priority of action** | **Timeframe for action** | **Responsible unit/person for action (specify person if possible)** | **Partners (specify unit/department or other stakeholders)** | **Steps required to fulfil action** |
| --- | --- | --- | --- | --- | --- | --- |
| **1** | **LEGAL** | | | | | |
| **1a** | Perform a full gap analysis of the legal framework to identify urgent needs for reform | High | Medium | Veterinary and Animal Health Directorate | Legal department of MOA |  |
| **1b** | Revise legal acts according to gap analysis and to Appendix IV | High | Long | Veterinary and Animal Health Directorate | Legal department of MOA | See Appendix IV |
| **1c** | Define a plan to strengthen the enforcement of existing laws with regards to 1) reporting system  2) data sharing  3) Private Veterinary Association  contribution to SyS | Urgent | Short | Veterinary and Animal Health Directorate | Legal department of MOA |  |
| **2** | **OPERATIONAL** | | | | | |
| **Stakeholders and data providers relevant for SyS** | | | | | | |
| **2a** | Engage the relevant stakeholders and data providers identified during the project (See Appendix II) | High | Short | Veterinary and Animal Health Directorate | JCDC, Legal department | 1. For each stakeholder/data provider, define the collaborative framework (e.g., contract, MoU, protocols etc.), also based on the PPP from WOAH^3^ 2. Finalize the agreed collaborative framework according to Appendix II |
| **2b** | Define a customized communication strategy to engage stakeholders and data providers based on Appendix II | Moderate | Short | Veterinary and Animal Health Directorate | JCDC |  |
| **2c** | Increase awareness of data providers on the SyS impact and expected benefits | High | Medium | Veterinary and Animal Health Directorate | JCDC | - Define awareness campaign plan - Develop educational materials as needed - Implement the campaigns |
| **2d** | Assess the effectiveness of the communication and awareness strategies | High | Long | Veterinary and Animal Health Directorate | JCDC | 1) Define criteria for evaluation |
| **Data / Data analysis** | | | | | | |
| **2e** | Confirm data streams to detect syndromes | Urgent | Medium | Veterinary and Animal Health Directorate | JCDC/University (JUST), External consultants | - Perform 1st statistical evaluation of identified data streams in Appendix III (data quality) - Prioritize data streams/syndromes from Appendix V |
| **2f** | Development of analytical algorithm (SyS statistical model) | Urgent | Medium | Veterinary and Animal Health Directorate | JCDC/University (JUST), External consultants | 1. Define ToR of SyS Statistical model 2. Develop and test the SyS stat model |
| **IT-EIDSS** | | | | | | |
| **2g** | Customization of EIDSS based on needs | High | Medium | Veterinary and Animal Health Directorate | B&W, External consultants | - Define needs - Customize EIDSS - Integration/link of SyS Statistical model in/to EIDSS - Testing/validation |
| **2h** | Define EIDSS management for SyS | Moderate | Medium |  |  | 1) Define support needed by host organization for EIDSSS (e. g. Black and Veatch) |
| **3** | GOVERNANCE | | | | | |
| **3a** | Policy discussion with MoA to confirm the support for SyS development | Urgent | Short | Veterinary and Animal Health Directorate |  |  |
| **3b** | Identify and address key aspects of data governance, in particular, legal issues on data sharing and data confidentiality | High | Medium | Veterinary and Animal Health Directorate | Legal department, Stakeholders and data providers representatives |  |
| **3c** | Set up a steering committee (SC) to oversee the implementation and management of the SyS and a technical committee for technical support, and empower their functions | High | Short | Veterinary and Animal Health Directorate | Identified members as in chapter 7.1 | 1. Define mandate and roles and coordination of the steering committee (SC) and technical committee (TC) 2. Develop TOR of main members of the steering committee and TC 3. Identify the main members of the SC and TC 4. Define a plan for regular meetings of SC and TC |
| **3d** | Define the procedures (i.e., SOPs) to react to alerts/alarms created by the SyS | High | Medium | Technical committee of the SC |  |  |
| **3e** | Agree on the financial support needed (for training, data analysist, IT-person) for SyS implementation from MoF | High | Medium | Veterinary and Animal Health Directorate | JCDC, MoF | 1. Define the sustainable finances/budget needed 2. Policy meeting with MoF |
| **4** | **CAPACITY BUILDING** | | | | | |
| **4a** | Implement technical trainings to enhance capacity for SyS implementation and management | High | Long/ Continuous | Veterinary and Animal Health Directorate | JCDC, University (JUST), External consultants, international agencies | 1. Define the capacity building needs for SyS 2. Define a training plan 3. Implement the trainings |
| **5** | **SYS EVALUATION AND NATIONAL UPSCALE** | | | | | |
| **5a** | Develop a framework for SyS evaluation | High | Long | Veterinary and Animal Health Directorate | JCDC, University (JUST), External consultants, international agencies | 1. Define the criteria for evaluating the success of the system 2. Define who will evaluate it 3. Develop the framework/tool for evaluation |
| **5b** | Develop a road to expand to other species/production sector and to upscale the SyS at National level | High | Long | Veterinary and Animal Health Directorate | JCDC, University (JUST), External consultants, international agencies, Other relevant stakeholders |  |
